# Surgical Cases Treated Homœopathically

**Published:** 1893-01

**Authors:** W. L. Reed

**Affiliations:** st. Louis, Mo.


### SURGICAL CASES TREATED HOMŒOPATHICALLY.

W. L. Reed, M. D., St. Louis, Mo.

A few years since, a young man came into my office apparently suffering great pain. Had his hand elevated before him, holding it with the other. When his hand hung down the pain was unendurable. He called for a basin of cold water, as bathing the hand afforded great relief. Had slept none for four nights. The pains were of a burning, stinging character, accompanied with chilliness. He had fallen asleep during the forenoon in a wagon-shed, being exhausted. He threw out his arm over his head. The sun fell upon it and instantly awoke him, with intense pain, requiring a cold bathing. He came to get his finger lanced. The forefinger of the right hand was greatly swollen, of a bluish red color. He said he had a felon. Now, as to what was the matter with this boy did not particularly concern me. An allopath, or a pathological homœopath would have at once split the finger with a lancet, to relieve the tension. Well, this would surely have done it, but have you, in such a procedure cured your patient? The cause that produced the felon has not been removed. The felon is only the product of a diseased condition of the organism. Is there anything scientific in treating the product of disease? I did not have to use a lancet, because I happened to be conversant with the pathogenesy of a drug that produces such similar symptoms upon a healthy organism. I commiserate the poverty of materia medica in a physician who fails to "catch on" in such cases. No intelligent physician has to resort to such barbarous procedures to combat disease. A knowledge of the pathogenesy of Pulsatilla is all that is necessary to afford complete and perfect relief. It is sufficiently clear to all present. I gave him one dose of Puls.$^{51m}$ dry, on his tongue. In twenty minutes, he said his finger felt better. In one hour and twenty minutes he left my office for home, his hand swinging by his side. No desire for cold applications nor any more pain. Went to bed and slept fifteen hours; awoke with his finger freely discharging laudable pus. Came to see me and had experienced no pain during the twenty-four hours after taking the medicine. I gave no more medicine, but plenty of S. L. Dressed the finger in absorbent cotton. He had a rapid recovery. He came to me in about six months with another felon developing on the forefinger of the left hand. The same symptoms were present. I gave two doses of Puls.$^{51m}$ (F.), which speedily aborted the whole affair. The last time I saw him, which was two years after, he had had no further trouble.

No. 2.—A lady called me to see her daughter, who was suffering very severely from a hard hot swelling on the palmar surface of one of her thumbs. She had not slept any for three nights. She was so cross they could scarcely live with her. No one could come near her or speak to her. Wanted to sit by the kitchen stove all the time and have the hand wrapped up warm; scolding any one who would open the door or walk heavily upon the floor. There was intense swelling with throbbing and fluctuation.

"Is it not a felon, Doctor?" says the mother; "does it not need to be opened?" "Yes; but she must have something to relieve the pain." The girl acquiesced with my statement, and gave her mother a scowl for suggesting such barbarous notions. "Let her take a teaspoonful of this medicine in the glass every half-hour or till she goes to sleep. Then stop till I see her again." At four P. M. she began taking the medicine, and by eight P. M. she was sound asleep. To this intelligent assembly it is needless to state that Nux-v.$^{200}$ was the remedy given. The pains all were speedily assuaged, and next morning it was discharging freely. The girl was sweetened up gloriously, to the infinite satisfaction of the whole family.

This is one of the many cases of a similar character that I have cured with Nux-vom. No occasion to resort to surgical measures for relief of the pain. One case I remember where there was a felon on each thumb with all the above symptoms speedily cured with Nux-vom.

No. 3.—A lady sent for me to call and see a "gathering," as she called it, in the axilla. She was obliged to hold up the arm constantly and sit by the stove. I found redness, heat, and swelling and extreme sensitiveness to *touch.* She could not bear to have a poultice applied. Been suffering for three days, with very little sleep. "Will it have to be opened?" she inquired. "Yes; but I shall not torture you by resorting to surgical measures. Take a teaspoonful of this medicine every hour till you sleep. She soon was relieved, and was freely discharging pus by nine o'clock P. M. The remedy in this case is also clear to a homœopathician. Hepar-sul. will nearly always be indicated in such cases.

The same afternoon, just across the street, another case of arm-pit boil, with the same symptoms, was speedily cured in one night's time with Hepar-sul. These cases were wonderful lessons to me, as I had just espoused the cause of Homœopathy. I was gloriously enthused and was obliged to be satisfied with glory, as that is all the pay I ever got for treating them.

There is a great deal of similarity between a Nux-vom. and a Hepar-sul. felon, and there is a striking dissimilarity. Both assail the palmar surface of thumb, Nux either thumb or both at the same time; seldom if ever the finger—at least, I have never seen the finger that required Nux. In Nux we have great over-sensitiveness to impressions upon the senses; noise, smell, light, music, other people's talking, draught of cold air, opening of doors. Both have irritability, but Nux is more marked; more vehement. In Nux there is very little sign of matter except a fluctuation. In Hepar there is yellow skin, the whole surface shows deposit of matter underneath the skin. Great sensitiveness to touch is the characteristic distinction—a throbbing pain in each; but the weight of the poultice cannot be endured in Hepar-sul. The right thumb is usually the one assailed, or one of the fingers in Hepar. Both desire heat and worse from a draught of air. In the *Guiding Symptoms* there is undoubtedly a grave error. See Nux. The text reads $<$ from warmth and warm water; should read $>$ from warmth and warm water and heat of bed, $<$ letting the limb hang down. This I have frequently verified.

The similarity between Puls. and Fluoric acid is very striking. Both require cold applications and both are often required in treating felons. Fluoric acid is more often required in simple onychia where cold applications relieve. Ledum also causes onychia from pulling off hangnails or a hurt, where cold also relieves. Fluoric acid, like Puls., usually attacks the first and second phalanx of index fingers. In Fluoric acid the pain is like that of Silicea, deep seated beneath the periosteum, and usually known as a bone felon. The finger usually swollen to four times its size, and in Fluoric acid opens on the dorsum of the finger, and exudes an ichorous, purulent discharge. Same as Puls., worse in the evening, finger usually of a pear shape in Fluoric acid.

Homœopaths ought to preach what they believe. "When right in it, put might in it." I might give many more such cases as here given; all of which have been very successfully treated by a strict observance of the law as transmitted to us by our fathers. We have received from our illustrious predecessors many evidences of the all-sufficiency of the law. No language is too strong, no logic too vigorous, no scholarship too thorough, no eloquence too rich and copious to convey the wonderful beauties of the efficacy of the simillimum to eradicate the disease.

I commiserate the ignorance of the physician who is obliged to resort to pathological prescribing to cure the sick. Are we losing the wisdom of our fathers? Ought we not to know more than they when our resources are so much greater? And now, my fellow-physicians, may I not make bold to affirm that the time has come when we ought to be progressive, to measure up to the demands of the age, to realize the brightest destiny of our glorious system of medicine. We must hail with sincere joy every honest and legitimate agency which promises to close up the ranks, and unify the aims and purposes and methods to disseminate the true principles of Homœopathy.

### Discussion.

Dr. Hitchcock—In this connection I wish to ask a question in regard to Juglans Nigra, the black walnut. A friend of mine, a carpenter, found he could never work in black walnut without the fingers and thumbs of both hands becoming covered with hangnails. Is there anything in the proving of Juglans Nigra having a bearing on that condition?

Dr. James—I want to enter a protest against the use of the crescendo and diminuendo mark in provings. Quite a number of errors have crept into our works through their use, through the marks getting reversed—a very easy thing for the printer to do. It is to the use of these marks that we owe the errors in *The Guiding Symptoms*, to which Dr. Reed refers.

